# 

**Published:** 1783

**Authors:** 


### P R O M O T E D.

Lately, Mr. Conrad Monch, apothecary at Caſſel, to be profeſſor of botany in the Caroline academy in that city.

*June* 12. Mr. James Simpſon to be ſurgeon to the Magdalen Hoſpital in London in the room of Mr. William Blizard, who reſigned.

*Oct.* 9. Charles Hawkins, Eſq. to be ſurgeon to the king's houſhold in the room of George Edward Hawkins, Eſq. deceaſed.——10. Mr. William Walker to be ſurgeon to St. George's Hoſpital in the room of G. E. Hawkins, Eſq.—Dr. Adair Crawford to be phyſician to St. Thomas's Hoſpital in the room of Dr. Henry Revel Reynolds, who reſigned.—31. Dr. Benjamin jamin Chandler, phyſician at Canterbury, to be an extra-licentiate of the Royal College of Phyſicians, London.

*Nov.* Mr. Benjamin Biggs, of the iſland of St. Croix, to be doctor of phyſic in the univerſity of St. Andrews.—15. Mr. John Connor to be ſurgeon to the General Hoſpital in the Leeward iſlands.——20. Alexander Monro, M. D. profeſſor of anatomy at Edinburgh, and John Hunter, Eſq. of London, F. R. S. to be foreign aſſociates of the Royal Academy of Surgery at Paris.

*Dec.* John Birch, Eſq. to be ſurgeon extraordinary to his R. H. the Prince of Wales.—4. Dr. John Marſhal to be fellow of the Royal College of Phyſicians at Edinburgh.—20. Lucas Pepys, of London, M. D. phyſician extraordinary to the King, to the dignity of a baronet of Great Britain.—22. Dr. Thomas Denman and Dr. William Oſborne to be licentiates in midwifery of the Royal College of Phyſicians, London.

---

### D I E D.

Lately, at Paris, Raymond de la Riviere, Francis Bidault, and Martin Nouguez, doctors

 regent regent of the faculty of phyfic in that city.— At Amfterdam, John Burmann, M. D. profeffor of botany.

1781. At Stutgard, Dr. W. Reufs, firft phyfician to the Duke of Wirtemberg.

1782. *March* 11. At Wittenberg, Dr. Geo. Auguftus Langguth profeffor of pathology and furgery.——17. At Bafil, in Switzerland, the learned Daniel Bernouilli, M. D. profeffor of phyfic and natural philofophy in the univerfity of that city, fellow of the R. S. of London, and of moft of the learned focieties in Europe. From his eulogium lately read before the Academy of Sciences at Paris, we learn, that he was born on the 9th of Feb. 1700, at Groninguen, where his father, John Bernouilli, a native of Bafil, was profeffor of mathematics, and celebrated for his fkill in that fcience. He was at firft intended for trade, but his father indulged his inclination for phyfic. At the age of 24 he vifited Italy, and the year following accepted an invitation from the Court of Ruffia, and fettled at St. Peterfburgh, where he remained till the year 1733, when he was nominated to a medical profefforfhip at Bafil. In the title of his firft work, '*Exercitationes quædam Mathematicæ*,' he ftiled himfelf fimply '*Daniel Bernouilli Joannis filius*;' and and this plain diſtinction he continued to adopt ever after, though loaded with literary honours. Nine times he obtained the premium given by the Academy of Sciences at Paris, and once, viz. in 1734, divided it with his father, who, inſtead of being pleaſed at ſeeing a ſon ſo worthy of himſelf, never forgave this undutiful competition as he termed it. The truth is, the ſon's piece was not only ſuperior to the father's, but he had eſpouſed in it the Newtonian doctrine, and his father was a rigid Carteſian. In 1748 he was elected one of the eight foreign aſſociates of the Academy at Paris in the room of his father. He himſelf is ſucceeded in this honour by his brother John, and it is a circumſtance very flattering to his family, that for the ſpace of eighty-four years, that is, ever ſince the firſt inſtitution of the claſs of foreign aſſociates, the name of Bernouilli has appeared in the liſt. He was never married, had the reputation of being a free thinker, and was ſaid to be fond of money; but he was ſo much revered by his fellow-citizens, that when he walked through the ſtreets all ranks of people were eager to ſhew their regard for him. He was remarkably temperate and uniform in his manner of living, and preſerved his health and his faculties unimpaired till he was nearly eighty years of age. After that he became infirm. In March 1782 his infirmities increafed, and he had only a painful exiftence. On the 17th of that month his fervant found him dead in his bed. By his will he has left an endowment for an inftitution at Bafil in favour of poor ftudents. He ufed to relate two anecdo es of himfelf, which he faid gave him more pleafure than all the literary honours he had acquired. One of thefe was his meeting with a man of learning in one of his journies, whofe curiofity being excited by the converfation of his fellow-traveller was induced to enquire his name. "I am Daniel Bernouilli"—faid he—with great fimplicity; and "I am Ifaac Newton," replied the other, thinking his new acquaintance was joking with him. Another time Koenig, a celebrated mathematician, dining with Bernouilli, was fpeaking to him of a difficult problem which had coft him a great deal of labour to folve. Bernouilli continued to do the honours of his table, and before they parted prefented Koenig with a more elegant folution of his problem than that which had occafioned him fo much trouble.

*May* 5. At Erfort, aged 45 years, Dr. William Bernard Tromfdorf, profeffor of phyfic.—

31. Dr.

Dr. Matthew Francis Alix, professor of physic and surgery at Fulda.

*August.* At Stockholm, Anthony Hoffman, M. D. physician in ordinary to the King of Sweden.—7. At Berlin, aged 74 years, Andrew Sigismond Marggraf, a celebrated chemical writer, director of the natural philosophy class of the Academy of Sciences at Berlin, and one of the foreign associates of the Academy of Sciences at Paris.

*Nov.* 29. The hon. Coote Molesworth, M. D. F. R. S. formerly physician to the garrison at Minorca. He was born in 1698, and was elected of the Royal Society in 1730.

1783. *January.* At Dresden, Charles Joseph Oehme, M. D.

*Feb.* 15, At Leipsic, of the small pox, in his 29th year, Frederick Andrew Gallisch, M. D. professor extraordinary of physic.

*March* 25. At Leipsic, Daniel Reichel, M. D. who succeeded Ludwig as editor of the Leipsic Commentaries, and who is himself succeeded in that office by Dr. N. G. Leske, professor of natural history.

*May* 22. At Wittemberg, aged 88, Dr. Daniel William Triller, professor of physic, whom Haller in his *Biblioth. Anatom.* stiles ‘ *vir eruditissimus,*

*tiſſimus, Græcarumque et Latinarum literarum peritiſſimus.*' His works were collected and publiſhed at Frankfort in 1772, in 3 vols. 4to. under the title of *Opuſcula Medica ac Medico-phyſiologica ab auctore recognita, aucta et emendata.*

*Aug.* At Paris, M. Pierre Morlanne, ſurgeon. —At Stalbridge in Dorſetſhire, Mr. Grimſtead, ſurgeon and apothecary.

*Sept.* 18. At Bourbonne-les-Bains, Ann Charles Lorry, doctor-regent of the Faculty of Phyſic, and V. P. of the Royal Medical Society at Paris. He was born at Crones, near Paris, Oct. 10, 1726. His father was Francis Lorry, profeſſor of Civil Law in the Univerſity of Paris, and author of a learned Commentary on Juſtinian's Inſtitutes. He received his claſſical education under Rollin, and ſtudied phyſic under Aſtruc and Ferrein. He was admitted of the faculty in 1748. His intenſe application to ſtudy brought on the gout very early in life. During the great cold in Feb. 1782, he had a paralytic ſtroke which deprived him of the uſe of his left ſide. The following ſummer he grew better, but afterwards relapſing, he determined to try the effects of the hot ſprings of Bourbonne. Dr. Hallé (his nephew) and the Abbé Teſſier accompanied him thither in Auguſt 1783. He was mild and gentle gentle in his manners, and poſſeſſed great activity of mind, a ſtrong memory, and extenſive erudition. His principal works are *Eſſai ſur les alimens*, 12mo. *De melancholia et morbis melancholicis*, 2 vols. 8vo. and *De morbis cutaneis*, 4to. He was likewiſe the author of ſeveral valuable papers in the memoirs of the Royal Medical Society. The following inſcription appears on his tomb:

Hic jacet

Præcipiti fato, nondum annis,

Dudum laboribus confectus,

Anna-Carolus Lorry, Pariſinus,

Doctor Medicus Pariſienſis,

Societatis Regiæ Medicæ, naſcentis columen,

Adultioris decus & ornamentum.

Integritatæ vitæ, amœnitate morum,

Ingenii acumine, incredibili doctrina

Laborum utilitate;

Pietate in Deum, amore erga ſuos,

Sedulitate apud ægros, benevolentia apud omnes,

Commendatus.

Thermas Borvonenſes, tot millibus ſalutiferas,

Inutiles expertus,

Flebilis multis,

Obiit

Obiit Borvonæ die xviii menfis Septembris,

Anno Domini M.DCC.LXXXIII,

Ætatis lvi, menf. xi, dieb. viii.

Quam viventi pacem contulit mens fibi bene confcia,

Eam defuncto concedat divina mifericordia.

—27. Mr. J. Randall, apothecary, in King-ftreet, Covent Garden.

*Oct.* At Paris, at a very advanced age, Ant. Nunes Ribeiro Sanchez, doctor of phyfic in the univerfity of Salamanca, and formerly firft phyfician to the emprefs of Ruffia, author of a learned effay on the origin of the venereal difeafe, and other works.—3. At Ripple-houfe, near Deal in Kent, aged 62, of a paralytic ftroke, Robert Lynch, M.D. phyfician at Canterbury, formerly fellow of Corpus Chrifti College, Oxford, and one of Dr. Radcliffe's travelling phyficians.—7. At St. Neot's in Huntingdonfhire, Mr. Vickery, furgeon and apothecary.—20. At Bievre le Chatel, M. Germain Pichaut de la Martiniere, Knight of the order of St. Michael, and firft furgeon to the late and prefent kings of France, in which poft he fucceeded the late M. de la Peyronie, in 1747.—30. At Nancy, Dr. Platel, confulting phyfician to the late king of Poland, and fecretary tary of the college of phyſicians of Lorraine.— At Morpeth, in Northumberland, Mr. Francis Laidman, ſurgeon and apothecary.

*Nov.* 1. Of a jaundice, aged 43 years, Charles Von Linné, M.D. profeſſor of phyſic and natural hiſtory, and ſuperintendant of the botanic garden at Upſal. He was the only Son of the illuſtrious naturaliſt of the ſame name lately deceaſed, and dying a batchelor, the male line of Linnæus is now extinct. About two years ago he viſited this country, and during his reſidence here was attacked with the diſeaſe which has ſince proved fatal to him. The public are indebted to him for a valuable Supplement to his father's *Syſtema Vegetabilium.*

*Dec.* 1. At Abingdon, in Berkſhire, aged 75, Mr. Graham, apothecary.

---

### SECTION IV.

### QUARTERLY CATALOGUE.

1. OBſervations on the nature, kinds, cauſes, and prevention of inſanity, lunacy, or madneſs. By *Thomas Arnold*, M. D. vol. I. containing obſervations on the nature and vari-

 ous ous kinds of infanity. 8vo. *Robinfon*, London, 1782. 324 pages. 5s.

The fecond volume of this performance is faid to be in the prefs, as foon as that appears we mean to give an account of the whole work.

2. Obfervations on the late Influenza, the *Febris Catarrhalis Epidemica* of Hippocrates, as it appeared at London in 1775 and 1782. By *William Grant*, M. D. 8vo. *Cadell*, London, 1782. 40 pages.

Hippocrates in the 7th book of his Epidemics, fect. 31, has defcribed a fpecies of catarrh which he fays was very general, but not fatal, although it lafted, in fome inftances, twenty days. He diftinguifhes it from the common catarrhous fever by the malignant fymptoms that attended it, viz. a remarkable ftupor, and attack on the head and nervous fyftem in the very beginning of the complaint. This defcription our author thinks is applicable to the epidemic catarrhs of 1775 and 1782. With regard to the firft appearance of the latter in London, Dr. Grant differs materially from our obfervations on the fubject (fee vol. 3. p. 203). We mentioned the beginning of May as the period at which it began to be felt here, and the

20th

20th of that month as the time when it became extremely general, but Dr. Grant dates its firſt appearance in March, and obſerves that by the middle of April it was ſpread all over London and its environs.

3. A letter to Dr. Leſlie, F. R. S. on the Influenza; as it appeared at Newcaſtle upon Tyne. By *John Clark*, M. D. 8vo. Newcaſtle, 1783. 26 pages.

Dr. Clark mentions ſeveral curious facts which clearly prove the influenza to have been propagated by contagion. He obſerves, that it firſt made its appearance at Shields, the port of Newcaſtle, on or about the 26th of May 1782, and was ſuppoſed to have been brought thither by the crews of ſome ſhips from London. The maſter of a veſſel who arrived at Shields 48 hours after he left the river Thames, came to his office on the 28th of May, labouring under the diſtemper. On the 29th one of the clerks in the office was ſeized, and as far as our author could learn, was the ſecond perſon who was attacked with the diſeaſe at Newcaſtle. In about ſix or eight days after the epidemic had appeared in that town the villages in the neighbourhood were attacked. About the 16th of June it began to abate, and in a week after totally disappeared.

4. Practical observations on amputation. By *R. Mynors*, surgeon. 8vo. *Robinson*, London, 12mo. 2s. 6d.

5. Enchiridion Botanicum, complectens characteres genericos et specificos plantarum per insulas Britannicas sponté nascentium, ex Linnæo aliisque desumptos. Edidit *Arthurus Broughton*, M. D. 8vo. *Robinson*, London, 1782. 226 pages. 4s.

6. An essay on the use of the red Peruvian bark in the cure of intermittents. By *Edward Rigby*, member of the corporation of surgeons in London. 8vo. *Johnson*, London, 1783. 107 pages.

7. A treatise on the infantile remittent fever. By *William Butter*, M. D. fellow of the royal college of physicians, and member of the medical society, both of Edinburgh, 8vo. *Robson*, London. 50 pages.

The author's account of the symptoms of what is commonly called the worm fever is accurate, and shews him to be a man of observation. What he says of the method of treatment in cases of this sort likewise deserves attention.

8. A

8. De la Pulmonie; de ſes ſymptomes, de ſes cauſes, et de ſa curation. *i. e.* Of Pulmonary Phthiſis, its ſymptoms, cauſes, and cure. By *Jeannet de Longrois*, doctor regent of the faculty of phyſic at Paris. 12mo. Paris, 1781. 207 pages.

9. *Joannis Hedwigii*, Societatum oeconomiæ Lipſienſis, et nat. ſcrutatorum Berol. memb. honor. Fundamentum Hiſtoriæ Naturalis Muſcorum frondoſorum, concernens eorum flores, fructus, ſeminalem propagationem, adjectâ generum diſpoſitione methodicâ, iconibus illuſtratis. 4to. Leipſic, 1782, in two parts, with ten coloured plates, and about 100 pages of letter preſs in each.

Dillenius and Linnæus were ſo unſucceſsful in their attempts to diſcover the fructification of Moſſes, that M. de Necker has actually denied the exiſtence of the ſexual organs and ſeed in theſe minute plants. The honour of diſcovering and deſcribing their flowers, their fruit, and their mode of propagation by ſeeds was reſerved for Mr. Hedwig.—In the firſt part of his work he deſcribes the inſtruments neceſſary for repeating his experiments. Good microſcopes and a few needles are requiſite for this purpoſe. The minute parts to be examined are to be placed placed in a drop of water, otherwise they would dry and crisp instantly. He finds that mosses have antheræ, pistils, organs of generation similar to those of perfect vegetables, and that they have often male and female powers distinct and separate, but it seems that those which have been hitherto mistaken for females by Linnæus and others, are male, and the supposed antheræ are capsules filled with seed. In some he has found two species of organs different from those requisite for generation. The use of these he is unable to ascertain. In the second part of his work he gives a definition of mosses, treats of their propagation by seed, and presents us with a new arrangement of their genera.

10. Beytrage zur Geschiete der frühlings-epidemie, &c. *i. e.* Additions to the history of the epidemic of the spring of 1782. By *J. D. Metzger*, aulic counsellor to the king or Prussia, doctor and professor of physic at Koningsberg. 8vo. Koningsberg, 1782. 80 pages.

The influenza began to prevail at Memel on the 4th, and at Koningsberg on the 15th of March. It proved fatal, we are told, only to a few aged and infirm people.

11. Peterburgische Kanzel-Vortræge, &c. *i. e.* Discourses delivered at Petersburgh. *By Joa-*

*chim-Chriſtian Grot*, rector of St. Catherine Waſiley-Oſtrow. 8vo. Leipſic, 1782. 440 pages.

We have here eleven ſenſible diſcourſes or ſermons in favour of inoculation. It appears that in the ſpace of three years 6796 perſons have been inoculated in a houſe erected for that purpoſe at Srkutzk, at the expence of the empreſs of Ruſſia.

12. Obſervationes, quibus præſtantiores vires Corticis Peruviani Rubri, in cura Intermittentium aliarumque febrium ſtabiliuntur. Accedunt hinc indé annotationes in curam aliorum morborum eodem remedio, authore *Gulielmo Saunders*, M.D. coll. reg. med. Londin. ſocio, noſocomii Guyani medico. Editio altera ex Anglico idiomate in Latinum verſa a *P. C. de Brabant*, med. Gand. 8vo. Gandavi, 1783. p. 124.

13. *Torberni Bergman* Chemiæ Prof. Upſal. et Equitis Aurati regii ordinis de Waſa, opuſcula phyſica et chemica, pleraque ſeorſim antea edita, jam ab auctore collecta, reviſa et aucta. Vol. III. 8vo. Upſal, 1783, p. 490.

For the contents of the two former volumes of this work we refer the reader to our 1ſt and 3d vols. The preſent volume contains the following lowing essays, viz. 1. *De analysi ferri*; 2. *De causa fragilitatis ferri frigidi*; 3. *De acidis metallicis*; 4. *De diversa phlogisti quantitate in metallis*; 5. *De stanno sulphurato*; 6. *De antimonialibus sulphuratis*; 7. *De productis vulcaniis*; 8. *De attractionibus electivis*; 9. *De ferro et stanno igne commixtis.*

14. Afhandling om Ben-Sjukdomar, efter erkande auctorers arbeten och anmoerkningar. *i. e.* A treatise on the diseases of bones, compiled from the works and observations of the best authors. By *Rowland Martin*, M. D. assessor of the royal college of physicians, and professor of anatomy and surgery. 8vo. Stockholm, 1782. 385 pages.

15. Sentimenti d'affetto e di riconoscenza degli studenti di medicina verso il loro immortale precettore il Sig. S. A. D. Tissot. 8vo. Pavia, 1783.

A collection of poems in honour of M. Tissot, by his pupils.

16. Abbildungen von arzneygewachsen, *i. e.* Plates of medicinal plants. 8vo. Norimberg, 1782.

This is the fourth number of the work. Each number contains fifty plants.

INDEX.

### I N D E X.

A.

Page

ACKERMANN, J. C. G. de dyfenteriæ antiquitatibus, — 331

Adanfon, M. on the gum trees of Senegal, — 28

Aikin, Jo. œconomiæ animalis delineatio, — — 104

Air, inflammable, proved to be phlogifton, — — 6

— fixed, its good effects, — — 74, 238

Alanfon, Edward, on amputation, — — 153

Aleppo, account of a fingular difeafe at, — — 366

Amputation, remarks on, — — 153, 273, 406

Armftrong, C. on the gonorrhœa of females, — — 206

Arnold, Dr. Tho. on infanity, — — 433

Afphaltites, lake, analyfis of its waters, — — 30

Affemblée publique de la S. R. de Montpellier, — 218

B.

Bark, Peruvian, natural and chemical hiftory of, — 304, 428

Barnades, Don Mich. principles of botany, — 322

Beard, growth of in a female, — — — 96

Benvenuti, Jo. della condizione de medici, — 110

Bergman, Sir Torb. analyfe du fer, — — 321

— opufcula phyfica et chemica, — 439

Bernouilli, Daniel, anecdotes of, — — — 426

Bew, George, cafe of a fhirt pin fwallowed with impunity, 77

Bladder, extravafation of blood into how to be removed, — 240

Bland, Dr. Robert, cafes of hæmaturia, — — 282

Blumenbach, J. F. Handbuch der naturgefchichte, — 331

Bones, fpecifically lighter than water, inftance of, — 309

Bordenave, M. on the motion of the ribs, — — 32

Brand, T. on ruptures, — — — 215

Broughton, Arthurus, enchiridion botanicum, — 436

Brugmans, feb. Juftin. lithologia groningana, — — 223

Bryan, Daniel, de cynanche typho, — — 327

Bryant, Charles, on two fpecies of lyncoperdon, — 104

— flora diætetica, — — 320

Butter, Dr. W. on the infantile remittent fever, — 436

Butts, Samuel de, de aeris effectibus, — — 327

C.

Cæfarean operation, cafes of, — — 94, 329, 378
Cambon, M. eloge de frere Cofme, — — 330
Camper, Dr. P. fur la meilleure forme des fouliers, — 343
—, on a new difeafe of the horned cattle, — 386
Cancer, remarks on, — — — 406

### INDEX.

Page

Cancerous breafts, improved method of amputating, —— 406
Cautery, applied to the head, fatality of, —— —— 353
Chambon, M. traitè de l'anthrax, —— —— 335
Cirillo, Dom. on the ufe of fublimate in frictions, —— 265
Clark, Dr. John, on the influenza, —— —— 435
Cold, extreme degree of determined, —— —— —— 205
Collection, academique, —— —— —— 335
Comparetti, And. de infirmitate nervorum, —— —— 224
Cook, Mr. cure of a fractured fcull, —— —— 72
Cornette, M. on the decompofition of neutral falts, —— 28, 30
Corp, Gulielm. de phthifi pulmonali, —— —— 326
Cofte, J. F. de antiqua medico-philofophia, —— —— 221
Cramer, G. C. P. de lichene iflandico, —— —— 223
Crell, Laur. on the acid of animal fat, —— —— 1
Creutzenfield, S. H. de Vigiliis Von, bibliotheca chirurgica, 323
Curtis, W. on the brown tail moth, —— —— 105

D.

Daignan, M. fur les fievres, —— —— 112

Dancer, Dr. Tho. expedition againft Fort St. Juan, —— 105

Deaths, —— —— —— —— 101, 211, 316, 425

Dijon, memoires de l'academie de, —— —— 379

Dictionnaire de fanté, —— —— —— 224

Doulcet, M. fur la fievre puerperale, —— —— 105

——————, tranflations of, —— 106

Dubourg, J. Barbeu, eulogy of, —— —— —— 40

Dyfury, chronic, remarks on, —— —— —— 69

E.

Epilepfy, remarkable cafe of, —— —— 76
Evory, Thomas, de febre puerperarum, —— —— 327
Experience in phyfic, treatife on, —— —— —— 9

F.

Fabre, M. fur differens points de phyfiologie, —— 336
Fearon, Henry, his method of amputating cancerous breafts, 406
Fenwick, Joan. Radulph, de plethora, —— —— 326
Fifcher, J. N. des Schielens, —— —— 223
Flores, Don Jofeph, fpecific for cancers, —— —— 323
Forfythe, Jacobus, de pneumonia, —— —— 327
Fothergill, Dr. John, life of, —— —— —— 176
Fourcroy, M. de, leçons elementaires de chemie, —— 332

G.

Galli, Angelo, adverfaria medica, —— —— 224
Garde, Henricus, de catarrho, —— —— —— 327
Gatterer, J. C. his meteorological obfervations, —— 341
Gerhard, C. A. des mineral reichs, —— —— 218
Gonorrhee, uber die behandlung der, —— —— 110
Gottingen, commentaries of the Royal Society at, —— —— 338
Gourlay, Gulielm. de eryfipelate, —— —— 326
Gout, medullary, hiftory of, —— —— —— —— 309
Grant, Dr. William, on the influenza, —— —— 434

Green,

### INDEX.

G. Page

Green, Mr. defcription of a lufus naturæ, — — 403

Grot, J. C. on inoculation, — — 439

H.

Haafe, J. G. cerebri hiftoria, — — 112

——, C. C. de caufis difficilis deglutitionis, — — 222

Haen, Anton. de, de vermibus et Ictero. — — 49

Hagen, C. G. hiftoria lichenum, — — 223

——, J. P. der praktifchen geburthyülfe, — — 329

Hair, maffes of found in the ftomach, — — 361

Hall, Harper, de melæna, — — 326

Halliday, Dr. W. his letter on the red bark, — — 416

Hamilton, Dr. Rob. cafe of a difeafed tefticle, — — — 172

Hare, Jacobus, de Syncope, — — 327

Harris, Dr. R. collectanea Hibernica medica, — 216

Hart, Edvardus, de morbis mammarum, — 326

Heart, ulcer of, — — — 358

Henry, Thomas, remarks on fermentation, — — 208

—— memoirs of M. de Haller, — 217

Henze, J. C. G. Verzeichnung Veteranifcher bucher, — 325

Hippocrates, his defcription of an epidemic catarrh, — 434

Hoffman, Tho. de præfagiis tempeftatis, — — 223

Holland, Philippus, de mente, — — 328

Home, R. on folvents, — — — 382

Humane Society, reports of, — — 216

Hunter, John, on the hearing of fifh, — — 229

Hutchinfon, Jac. de febrium mutatione, — — 328

Hydrocephalus, internus, remarks on, — — 78, 393

I.

Janffens, J. F. on fixed air in putrid fevers, — — 74

Ice water, good effects of in fchirrus, — — 350

Influenza, tracts relative to, — — 434, 435, 438

Ingenhoufz, Dr. J. on the influence of vegetables on animals, 233

Innes, James Dunbar, on the venereal difeafe, — 320

Inoculation, difcourfes on, — — 438

Julianus, A. de refina elaftica, — — 108

K.

Kirwan, Richard, on the fpecific gravities of falts, — 5

Kleine, medinifch chirurgifch abhandlungen, — — 336

Knight, Francis, effects of a large dofe of fugar of lead, — 286

L.

Labour, theory of, — — — 355

Laicharting, J. N. von, der Tyroler infekten, — 322

Lammerfdorf, Jo. Ant. de filicum fructificatione, — 112

Languedoc, epidemic of, defcribed, — 53, 107, 108

Laroche, M. de, fur la fievre puerperale — — 328

Laffone, M. de, on faline combinations of iron, — 27

Lavoifier, M. de, on the nature of acids, — — 35

—— heat, — — — 235

—— his analyfis of water, — — 414

Lauth, Tho. de analyfi urinæ, — — 220

Page

Lefebure, J. B. his edition of the aphoriſms of Hippocrates, — 223

Leſke, N. G. von dem drehen der ſchafe, — — 111

Lettſom, Dr. J. C. his life of Dr. Fothergill, — — 214

Leuthner, J. N. A. paſtoral arzneykunſt, — — 324

Linnæus, anecdotes of, — — 26

Lithotomy, performed at two different times, — — 242

Liver, caſe of a dropſy of, — — — 44

Longrois, J. de, ſur la pulmonie, — — 437

Lorry, M. obſervations by, — — 43, 371

——— anecdotes of, — — 430

Luſus Naturæ, curious one deſcribed, — — 403

M.

Macbride, Dr. David, eulogy of, — — 39

Malouin, M. eulogy of, — — — 24

Maret, M. on the conſtruction of hoſpitals, — — 380

Marſeilles, population and diſeaſes of, — — 369

Marſhall, And. de militum ſalute, — — 328

Martin, Rowland, inſtitutiones neurologicæ, — — 324

——— on diſeaſes of the head, — — 440

Maſturbation, curious caſe of, — — — 302

Membrana pupillaris, remarks on, — — 359

Menses Retentio — 339.

Mercury, its freezing point aſcertained, — — 205

Metzger, J. D. on the influenza, — — 438

Michell, Jan Peterſon, de ſynchondrotomia, — — 374

Microſcopic deception, inſtances of, — — 114, 115

Millar, Dr. J. his reply to Dr. Monro, — — 215

Milner, Dr. Tho. experiments in electricity, — 320

Mittie, J. S. ſuite de l'aitiologie de la ſalivation, — 334

Mochſen, J. C. W. der Wiſſenchafften, &c. — 111

Monro, Dr. Alex. on the nervous ſyſtem, — — 113

Monſter, deſcription of, — — 2

Montpellier, hiſtoire de la S. R. de, — — 109

Moſſes, their ſexual organs diſcovered, — 437

Murray, J. And. rare plants deſcribed by, — — 338

N.

Neuforn, J. C. S. von, de uſu cantharidum, — — 224

News, medical and philoſophical, — 88, 203, 299, 412

Nicholas, M. des epidemies de Dauphiné, — 334

Nobleville, Louis D. Arnold de, eulogy of, — — 38

O.

Oeſophagus, ſchirrus of, — — 45, 46

Opium, its efficacy in lues venerea, — — 429

Oſborn, Dr. William, on laborious parturition, — — 135

P.

Panzer, G. W. F. obſ. botanic, ſpecimen, — 107

Paris, memoirs of the R. Med. Soc. at, — 38, 148, 238, 358

——— Acad. of Sciences, at, — 24

Park, H. his new method in diſorders of the joints, — 273

Paton, Geo. de Typho petechiali, — — 326

Paulet,

### I N D E X.

Page

Paulet, M. on a fpecies of anthrax, — — 48

Percival, Dr. mifcellaneous facts and obf. by, — — 56

Perfect, W. cafes in midwifery, — — 216

Perrolle, M. Differt. anatomico acouftique, — 218

Pieces intereffantes fur la medecine, — — — 331

Plague, remarks on, — — — 364

Plants, medicinal, plates of, — — 440

Platina, effays on, — — — — 325

Pluvinet, B. N. de fermentatione, — — — 220

Pouteau, M. œuvres pofthumes, — — — 349

Prize queftions, — — 88, 203, 299, 300, 204, 413

Promotions, — — — 100, 210, 314, 424

Ptofis, or fall of the eye-lids, caufe of, — — 340

Pui, Simon du, de homine dextro et finiftro, — 52

Pylorus, fchirrous, cafes of, — — — 93

Q.

Quengfy, G. P. de, fur l'œil, — — — 219

R.

Raccolta di opufculi fifico-medici, = — — 109
Renny, G. on the venereal difeafe, — — 245
Renwick, W. on the medical fervice of the Royal Navy, — 319
Ribs, motion of in refpiraion, — — — 32, 33
Richter, A. G. chirurgical obfervations by, — — 339
Rigby, Edward, on the red bark, — — 436
Rollo, John, on preferving health in the W. Indies, — 104
Rofa, Michela, fopra alcune curiofita, — — 267
Rouffeau, L. Von dem Salze, — — 221
Rouffet, H. F. A. fur la petite verole, — — 324
Rymer, James, on the fection of the fymphyfis pubis, — 217

S.

Sabatier, M. obfervations by, — — 32, 33
Sage, M. on the red mines of copper, — — 31
Sanden, Dr. Tho. on fome fymptoms of fever, — 289
Saunders, Dr. W. obfervations on the red Peruvian bark, 318, 439
—— —— confirms the utility of opium in the lues venerea, 420
Saucerotte, M. concernant les femmes enceintes, — 328
Sayers, And. de menorrhagia, — — 326
Schæffer, J. U. G. der theoretifchen Arzneykunfte, — 323
Senfft, A. gefundheits katechifmus, — — 110
Sheldrake, T. remarks on Mr. Brand, — — 215
Shoes, the beft form of, — — — 343
Siebold, C. G. de parotide fchirrofa, — — 220
Simmons, Dr. on the natural hiftory of Peruvian bark, — 304
—— — fur le traitement de la Gonorrhée, — 321
Sivry, M. obfervations mineralogiques, — — 221
Small-pox, complicated with meafles, inftance of, — 301
Solvents, their efficacy and fafety afcertained, — 382
Spielman, J. R. pharmacopeia generalis, — — 264
Stevenfon, Dr. W. on Dr. Lee's gouty cafe, — — 104
Stokes, Jon. de aere dephlogifticato, — — 327

Suette

Page

Suette of Picardy, what, — — — 54

Swieten, Baron van, opera posthuma, — — 145

Sylvestre, P. de metastasi lactis, — — 224

Synchondrotomia, Jan. Peterson Michell, de, — 374

T.

Thomassin, M. sur le charbon malin, — — 336

Thunberg, C P. de gardenia, — — 224

Tissot, M. verses to, — — — — 440

Torlese, John, on a monstrous birth, — — 440

Transactions, Philosophical, — — 1, 225

Trinder, Dr. W. M. on the mineral waters of Essex, — 218

Troja, M. malattie degli occhi, — — — 220

U.

Ulcers of the legs, remarks on, — — 254

Underwood, M. on ulcers of the legs, — — ibid.

Uterus, imperforated, case of, — — 243

Uva Ursi described, — — — 264

V.

Vagina, obstruction of from a variolous ulcer, — 340

Vaughan, Ricardus, de rheumatismo, — — 328

Venereal disease, treatises on, — 110, 216, 245, 320, 321

Venom, a contagious disorder of the horned cattle so called, 386

Vicq d'Azyr, observations by, — 32, 42, 359, 372

Vitman, P. D. F. istoria erbaria degl' Alpi, — — 111

Vollstoendiges verzeichneiss aller gewoesche Deutschlands, — 322

W.

Water, proved to be not a simple element, — — 414

Watson, Rev. Dr. chemical essays by, — — 105

Weber, J. A. der thienschen feuchtigkeiten, — — 221

Wedgewood, J. his measure of the higher degrees of heat, 225

White, C. his account of Captain M——'s case, — 159

—— Dr. W. on the bills of mortality at York, — — 2

Wier, Edw. remarks on hydrocephalus, — — 78, 393

Willan, Dr. R. his account of a new species of byssus, 421

Wolstein, J. uber die viesuchen in Oesterreuk, — 112

Wooffendale, R. on the teeth, — — — 320

Z.

Zimmermann, Dr. on experience in physic, — — 9

